# A 5-min Cognitive Task With Deep Learning Accurately Detects Early Alzheimer's Disease

**DOI:** 10.3389/fnagi.2020.603179

**Published:** 2020-12-03

**Authors:** Ibrahim Almubark, Lin-Ching Chang, Kyle F. Shattuck, Thanh Nguyen, Raymond Scott Turner, Xiong Jiang

**Affiliations:** ^1^Department of Electrical Engineering and Computer Science, Catholic University of America, Washington, DC, United States; ^2^Department of Information Technology, College of Computer, Qassim University, Buraydah, Saudi Arabia; ^3^Department of Neuroscience, Georgetown University Medical Center, Washington, DC, United States; ^4^Department of Neurology, Georgetown University Medical Center, Washington, DC, United States

**Keywords:** Alzheimer's disease, machine learning, artificial neural networks, inhibition of return, neuropsychological test

## Abstract

**Introduction:** The goal of this study was to investigate and compare the classification performance of machine learning with behavioral data from standard neuropsychological tests, a cognitive task, or both.

**Methods:** A neuropsychological battery and a simple 5-min cognitive task were administered to eight individuals with mild cognitive impairment (MCI), eight individuals with mild Alzheimer's disease (AD), and 41 demographically match controls (CN). A fully connected multilayer perceptron (MLP) network and four supervised traditional machine learning algorithms were used.

**Results:** Traditional machine learning algorithms achieved similar classification performances with neuropsychological or cognitive data. MLP outperformed traditional algorithms with the cognitive data (either alone or together with neuropsychological data), but not neuropsychological data. In particularly, MLP with a combination of summarized scores from neuropsychological tests and the cognitive task achieved ~90% sensitivity and ~90% specificity. Applying the models to an independent dataset, in which the participants were demographically different from the ones in the main dataset, a high specificity was maintained (100%), but the sensitivity was dropped to 66.67%.

**Discussion:** Deep learning with data from specific cognitive task(s) holds promise for assisting in the early diagnosis of Alzheimer's disease, but future work with a large and diverse sample is necessary to validate and to improve this approach.

## Introduction

Alzheimer's disease (AD) is a progressive neurodegenerative disorder and the most common cause of dementia in older adults. Due to significant progress in basic and clinical research, putative disease-modifying treatments for AD may be on the horizon - which may be most effective in early disease stages. As a result, there is increasing impetus to develop techniques that have high sensitivity and specificity to assist in the diagnosis of early AD (Fiandaca et al., 2014).

Machine learning—with the ability to extract features from high dimensional spaces—holds strong promise in assisting disease diagnosis in both translational research and clinical practice (Weng et al., 2017; Dwyer et al., 2018), especially with recent advances in deep learning techniques (Esteva et al., 2017). Over the past decade, there has been increasing interest in developing machine learning techniques to assist in the diagnosis of AD and mild cognitive impairment (MCI) and to predict disease progression. Most of these studies focus on brain imaging data from magnetic resonance imaging (MRI) or positron emission tomography (PET) scans (Pellegrini et al., 2018), or cerebrospinal fluid (CSF) proteomics to assess CNS amyloid deposition (A), pathologic tau accumulation (T), and neurodegeneration (N) – the A/T/N criteria under the current NIA-AA research framework (Jack et al., 2018). Compared to brain imaging data, behavioral data are feasible and relatively inexpensive to collect. Behavioral data from speech (Fraser et al., 2016; Nagumo et al., 2020), body movement (Khan and Jacobs, 2020), and neuropsychologic test scores (Lemos et al., 2012; Williams et al., 2013; Kang et al., 2019; Lee et al., 2019a) may provide useful features to machine learning classifiers for the diagnosis of MCI and AD.

In addition to standard neuropsychological tests that are widely used in both research and clinical environments, cognitive tasks are usually highly specific and customized and are often only used in research studies. Compared to standard neuropsychological tests, cognitive tasks have certain advantages and disadvantages: on the one hand, cognitive tasks are usually limited by a lack of standardized data and/or validation with a large population of participants; on the other hand, cognitive tasks are often based on cutting-edge research hypothesis and may be more sensitive in detecting very specific changes in brain function due to brain disease such as AD (Perry and Hodges, 1999) – which might eventually lead to the development of improved and/or novel neuropsychological tests (or being integrated with existing neuropsychological test battery) that can be used in clinical practice after validation. Machine learning studies have shown that data from certain cognitive tasks may contain useful information to differential AD/MCI patients from healthy controls (Wallert et al., 2018; Valladares-Rodriguez et al., 2019; Hong et al., 2020). Therefore, it is of a high interest to investigate whether a combination of neuropsychological tests and cognitive task(s) may improve machine learning-based classification accuracy in AD (Wallert et al., 2018; He et al., 2019). In a previous study with traditional machine learning models and multivariate feature selection techniques, we investigated the classification performance with data from a standard neuropsychological test battery, a 5-min cognitive task, or both, to distinguish CN from MCI/AD patients (Almubark et al., 2019). The cognitive task was designed to assess the effects of spatial inhibition of return (IOR). Spatial IOR refers to the phenomenon by which individuals are slower to respond to stimuli appearing at a previously cued location compared to un-cued locations when the stimuli onset asynchrony (SOA) between the target and cue is long (~300–500 ms or more) (Klein, 2000). First reported by Posner and Cohen (1984), spatial IOR has been extensively studied, including in healthy older adults (Hartley and Kieley, 1995), patients with various neurogenerative disorders (Possin et al., 2009; Bayer et al., 2014), and non-human subjects (Shariat Torbaghan et al., 2012). In addition to the superior colliculus (Posner et al., 1985), cortical areas such as the temporoparietal junction (TPJ) and the inferior parietal cortex are important to maintain normal spatial IOR function (Seidel Malkinson and Bartolomeo, 2018; Satel et al., 2019). Both regions have been are implicated in AD progression (Besson et al., 2015), suggesting that spatial IOR may be useful to assist MCI and AD diagnosis. While early studies suggest that spatial IOR is relatively preserved in AD (Amieva et al., 2004), recently we (Jiang et al., 2020) and others (Tales et al., 2005, 2011; Bayer et al., 2014) have provided evidence that spatial IOR impairment in MCI/AD, and spatial IOR impairment in MCI patients may be predictive of conversion to dementia (Bayer et al., 2014). Therefore, machine learning with spatial IOR data may be useful in assisting diagnosis of MCI and AD. In addition, spatial IOR have two appealing features: first, the task is simple to understand and easy to implement, thus making it a feasible tool with AD/MCI patients in a typical clinical setting; second, spatial IOR is robust and resistant to practice effect (Pratt and McAuliffe, 1999; Bao et al., 2011), thus making it an ideal tool in longitudinal studies or clinical trials. However, in the previous study, we found that the classification performance with IOR data as well as the NP data had a low sensitivity and combining IOR and neuropsychological data did not significantly improve classification accuracy (Almubark et al., 2019), suggesting a need for further research.

Deep learning has advantages over machine learning due to its capacity of extracting useful features from highly complex and non-linear datasets (Pedregosa et al., 2011; LeCun et al., 2015), and is gaining popularity in AD research. For example, a PubMed search revealed 8 relevant publications before 2017, 8 in 2017, 26 in 2018, and 65 in 2019. Convolutional-Neural Network (CNN) is the most commonly used deep learning techniques (Gautam and Sharma, 2020). The overwhelming majority of these studies have been focusing on complex and high dimension brain imaging data, especially PET and structural MRI (Jo et al., 2019; Ebrahimighahnavieh et al., 2020; Gautam and Sharma, 2020; Haq et al., 2020). Several recent studies have aimed to integrate multimodal imaging to improve classification performance (Suk et al., 2014; Lu et al., 2018; Huang et al., 2019; Punjabi et al., 2019; Zhou et al., 2019). Deep learning can also help to identify features that are important for disease progression or serve as markers for clinical trials (Ithapu et al., 2015). In addition to harvesting brain imaging data [especially the multimodality imaging data from the public ADNI database (http://adni.loni.usc.edu/)], deep learning has been applied to biospecimens (Lee et al., 2019b; Lin et al., 2020), electronic health records (Landi et al., 2020; Nori et al., 2020), speech (Lopez-de-Ipina et al., 2018), neuropsychological data (Choi et al., 2018; Kang et al., 2019), and a combination of MRI and neuropsychological data (Qiu et al., 2018; Duc et al., 2020). By contrast, few studies have applied deep learning to cognitive task data, which – by design – is supposed to be more sensitive to detect early and mild neurocognitive impairment (Locascio et al., 1995; Perry and Hodges, 1999). Highly relevant to the present study, Rutkowski et al. applied various traditional and deep learning models to behavioral data collected from a facial emotion implicit short term memory task (Rutkowski et al., 2020). In their study, Rutkowski et al. obtained an accuracy close to 90% in distinguishing MCI from normal older adults with either deep learning or logistic regression, supporting a potential of deep learning with cognitive task to aid MCI/AD diagnosis. However, one limitation of their study was that the MCI status was solely defined by the Montreal Cognitive Assessment (MoCA) score rather than a formal clinical evaluation, which is necessary to diagnose MCI (Albert et al., 2011).

In the present study, we further investigated the classification performance of MCI/AD vs. CN using behavioral data from standard neuropsychological tests, a cognitive task (spatial IOR), or both. Both MCI and AD patients were formally diagnosed by clinicians with the consensus guidelines (Albert et al., 2011; McKhann et al., 2011). A variety of machine learning algorithms were tested: four traditional machine learning models and a feed-forward artificial neural network (ANN) model, which has been widely used in AD research (Jo et al., 2019).

## Materials and Methods

Machine learning was carried out using *Python* and related libraries including *Scikit-learn, Pandas, Numpy, TensorFlow*, and *Keras* (Pedregosa et al., 2011; Chollet, 2015). All experiments were conducted in Google Colaboratory platform (Bisong, 2019).

### Data

#### Participants

Twelve individuals with MCI, 16 individuals with mild AD, and 50 CN participated in the study between 2014 and 2015 (Table 1). The MCI and AD patients were part of the Memory Disorders Program cohort at Georgetown University Medical Center (https://memory.georgetown.edu/). There was no biomarker data from the majority of MCI subjects in this study. All MCI and AD participants were clinically evaluated by clinicians in the Memory Disorders Program with expertise and experience with MCI and AD research. The diagnosis for MCI was based on the clinical interview with the patients and their knowledgeable partners (and also with neuropsychological data when available) using the consensus criteria (Albert et al., 2011) – a Clinical Dementia Rating (CSR) score of 0.5 (https://knightadrc.wustl.edu/cdr/PDFs/CDR_Table.pdf). The AD diagnosis was based on clinical and biomarker data (if available) following the consensus guideline (McKhann et al., 2011). All healthy CN were recruited from the Washington DC metropolitan area. Prior to enrollment, a signed informed consent form approved by the Georgetown University Medical Center's Institutional Review Board was obtained from all participants and their legally authorized representatives (if they had a diagnosis of MCI or mild AD). With the entire study sample, the MCI/AD patients were significantly older than the CN (*p* = 0.0001). As the difference in age could potentially confound the classification results, we identified a subset of demographically comparable subjects, which include 16 MCI/AD patients (8 MCI and 8 AD) and 41 CN. The results from the demographically comparable subset of subjects are included in the main article, and the results from the entire study sample are included in the Supplementary Materials.

**Table 1:** The demographics and neuropsychological test scores of CN and MCI/mild AD participants.

**Characteristics**	**Entire study sample dataset**			**Demographically comparable dataset**		
	**CN**	**MCI/mild AD**	***p* ^b^**	**CN**	**MCI/mild AD**	***p* ^b^**
N (F)	50 (32F^a^)	28 (10F^a^)	n.s.^c^	41 (26F^*a*^)	16 (6F^*a*^)	n.s.^c^
Age	65.9 ± 6.2	72.7 ± 7.4	0.0001	67.4 ± 5.3	69.9 ± 5.3	n.s.
Education (years)	18.1 ± 3.9	18.2 ± 3.9	n.s.^c^	18.4 ± 3.4	19.0 ± 4.5	n.s.
%CA	82.0%	89.3%	n.s.^c^	78.1%	81.3%	n.s.^c^
MMSE	29.4 ± 1.0	25.8 ± 4.5	5.3E-06	29.3 ± 1.0	27.3 ± 2.3	3.0E-05
MoCA^d^	25.3 ± 1.8	21.0 ± 4.4	9.7E-05	25.2 ± 1.8	21.4 ± 4.4	0.0035
LM immediate	12.4 ± 3.6	6.6 ± 3.8	4.6E-09	12.0 ± 3.5	7.8 ± 4.2	2.9E-03
LM delayed	9.8 ± 4.3	4.1 ± 3.9	1.2E-07	9.5 ± 4.2	5.4 ± 4.3	0.0019
ADAS-cog	5.9 ± 3.4	18.7 ± 9.7	1.6E-12	6.3 ± 3.4	15.7 ± 7.7	3.1E-08
NPI^e^	2.0 ± 4.5	7.6 ± 8.8	0.0005	2.3 ± 4.9	5.1 ± 5.6	n.s.
LADL^e^	76.4 ± 2.8	67.4 ± 11.9	3.3E-6	76.3 ± 3.0	71.1 ± 8.5	0.0013
LVF	46.8 ± 12.9	37.5 ± 15.5	0.0058	48.2 ± 13.1	39.6 ± 14.0	0.035

*CA, Caucasian-Americans; MMSE, Mini-Mental State Exam; MoCA, Montreal Cognitive Assessment; LM, Logical Memory Test; ADAS-Cog, Alzheimer's Disease Assessment Scale-Cognitive subscale; NPI, Neuropsychiatric Inventory; LADL, Lawton Instrumental Activities of Daily Living Scale; LVF, Letter Verbal Fluency (Controlled Oral Word Association Test, COWAT)*.

a *female*;

b *uncorrected p-values for the difference between CN and MCI/mild AD with two-tailed two-sample t-tests (unless otherwise specified)*;

c *Fisher's Exact Test*;

d *MoCA were only administered to a subset of participants, including 22 CN and 22 MCI/mild AD patients form the original dataset and 17 CN and 11 MCI/mild AD patients from the demographics matched dataset, and MoCA test scores were not used in classification*.

e *NPI and LADL data was missing from one control and one MCI/mild AD patient*.

*The performance with the optimal hyper-parameter tuning for each dataset is shown in bold and italics font (optimal values for class weight and threshold)*.

#### Neuropsychological Data

The following neuropsychological data were collected from all participants: Mini-Mental State Examination (MMSE); Alzheimer's Disease Assessment Scale – Cognitive subscale (ADAS-Cog); F-A-S Letter Verbal Fluency (LVF) [or Controlled Oral Word Association Test (COWAT)]; Logical Memory subtest of the Wechsler Memory Scale (WMS) – fourth edition (WMS-IV). In addition, data with the Lawton Instrumental Activities of Daily Living Scale (LADL) and Neuropsychiatric Inventory (NPI) were collected and included in this study, as behavioral disturbance and loss of daily functioning are common in AD patients and can be assessed by these two tests (Cipriani et al., 2020; Cummings, 2020).

#### Cognitive Task [Spatial Inhibition of Return (IOR)]

**Figure 1:**
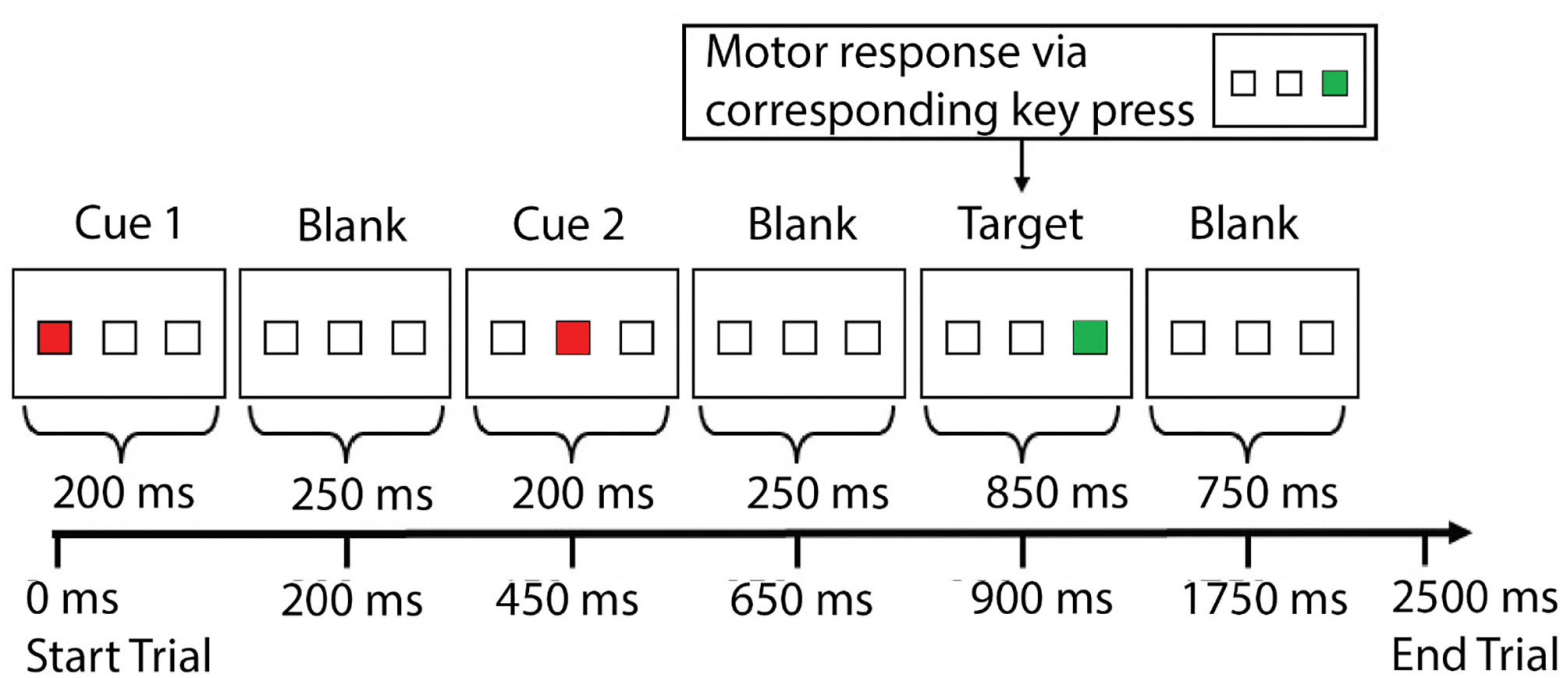
The cognitive task [spatial inhibition of return (IOR)] experiment paradigm. Within each trial, there were three sequentially presented visual stimuli—two cues (solid red square) and one target (solid green square)—with a blank screen in between. The three stimuli were presented serially. The two cue stimuli could appear in any of the three locations (left, middle, right), whereas the target stimuli could only appear in one of the two locations (left or right, but not the middle). Subjects were instructed to respond to the target (solid green square) by pressing one of two buttons in the right hand to indicate whether the target was presented at the left or right location (with the index finger or the middle finger). The two cues were presented 200 ms each, with a 250 ms break in between. The second cue was followed by another 250 ms break before the onset of the target, which was presented for 850 ms. The next trial started 750 ms after the offset of the target stimulus. Subjects had to respond within the 1.6 s time-window (before the onset of next trial). There were five conditions based on the relationship of the locations in which the three stimuli were presented: ***aaa***, in which the two cues and the target were presented at the same location; ***abb***, in which the second cue and the target were presented at the same location, and the first cue was presented at a different location; ***aba***, in which the first cue and the target were presented at the same location, and the second cue was presented at a different location; ***aab***, in which the two cues were presented at the same location, and the target was presented at a different location; ***abc***, in which the two cues and the target were presented at three different locations. The behavioral data from the study team can be found elsewhere (Jiang et al.), which includes detailed data from each individual subject that can be downloaded by other teams to test with their approaches. Note: ms, millisecond.

Experimental details can be found elsewhere (Jiang et al., 2020) and in Figure 1. Each trial lasted 2.5 s, and there were 130 trials in total (325 s).

### Experimental Design for Classification

The data described in the previous section was arranged into five datasets: Dataset 1 (NP), the scores of nine standard neuropsychological tests (including general cognitive assessments, learning and memory, language, and activities of daily living); Dataset 2 (IOR_trial_), the responses and reaction time of each trial from the 5.5-min spatial attention IOR task (for a trial without response, the values were set to 0 for response and 10,000 for reaction time); Dataset 3 (IOR_cond_), the overall accuracy of all trials (with non-responding trials included or excluded), the accuracy (with non-responding trials included or excluded), and the mean reaction time (correct trials only) of five experimental conditions (Figure 1); Dataset 4 (NP + IOR_trial_), a combination of Datasets 1 (NP) and 2 (IOR_trial_); Dataset 5 (NP + IOR_cond_), a combination of Datasets 1 (NP) and 3 (IOR_cond_). There were a total of 9 features in NP Dataset (7 neuropsychological tests, 3 scores for LVF test (the number of words generated from the three letters, F, A, and S, within 60 s for each letter, respectively, i.e., F: fruit, fog, fun, figure, etc.), 260 features for IOR_trial_ Dataset (responses and reaction time of 130 trials), 17 features for IOR_cond_ Dataset (accuracy and reaction time of experimental conditions, Figure 1), 269 features for NP + IOR_trial_ Dataset, and 26 features for NP + IOR_cond_ Dataset.

### Data Pre-processing

There were 4 missing values in Dataset 1 (NP) (see Table 1), 0 missing value in Dataset 2 (IOR_trial_), 0 missing value in Dataset 3 (IOR_cond_), 4 missing values in Dataset 4 (NP + IOR_trial_), and 4 missing values in Dataset 5 (NP + IOR_cond_). Missing values in data were handled first by using the multivariate imputation methods available in the fancyimpute library (Rubinsteyn, 2020). Four different missing value imputation techniques were tested: (1) Mean imputation fills a missing value with the mean value of the respective feature from the same group. (2) Nearest neighbor imputations weights samples using the mean squared difference on features for which observed data is contained in both rows. (3) Softimpute, fast and effective for datasets with high dimensionality, completes matrices through iterative soft thresholding of SVD decompositions (Mazumder et al., 2010). (4) Nuclear norm minimization adopts the *cvxpy* library (Diamond and Boyd, 2016) to provide a simple implementation of Exact Matrix Completion via Convex Optimization (Candès and Recht, 2009). Analyses were carried out using each of the four imputation methods, and nuclear norm minimization was chosen as it consistently provided superior results across all classification algorithms.

Imbalanced classes are common but may result in biased classifiers with poor accuracy on the minority class. To control for class imbalance in the present study (41 CN vs. 16 MCI/AD), an over-sampling technique called Synthetic Minority Over-sampling Technique (SMOTE) (Chawla et al., 2002) was used. Through over-sampling the minority class, the SMOTE technique has shown to improve classification accuracy of imbalanced datasets.

Feature scaling was also performed on both neuropsychological tests and cognitive task datasets. We used the standard scalar, which transforms the data to have the mean of zero with a standard deviation of one. Before the training, a grid search was conducted to obtain an optimal set of hyper-parameters for each algorithm. The grid search works by exhaustive searching through a specified subset of hyper-parameters, and find the best combination of parameters for each algorithm (Bergstra and Bengio, 2012).

### Traditional Machine Learning Algorithms

#### Algorithms

There are many supervised machine leaning algorithms for classification problems. Based on the size, quality, and nature of our data, four machine learning algorithms were investigated; Support Vector Machine (SVM), Random Forest (RF), Gradient Boosting (GB), and AdaBoost (AB) classifiers.

Support Vector Machines (SVM) are supervised machine learning algorithms that analyze data used for classification, regression and outlier detection (Cristianini and Shawe-Taylor, 2000). Linear SVM seeks a hyperplane that best separates two classes. SVM trains data to find multiple support vectors, which define the hyperplane. The prediction only relies on the support vectors. In addition to linear classification, SVM can use kernels to perform a non-linear classification by mapping their inputs into higher dimensional feature spaces.

Random Forest (RF) algorithm is an ensemble classifier consisting of many decision tree classifiers. Output is determined by the majority vote among all the decision trees for each sample (Breiman, 2001). The RF algorithm combines bootstrap aggregation (bagging) (Breiman, 1996) and random feature (Amit and Geman, 1997) to construct a collection of decision trees exhibiting controlled variation. Because the classification is not based on one tree alone, RF is thought to be more robust than a single decision tree classifier in performance.

Gradient Boosting (GB) is a machine learning technique for classification and regression problems that produces a prediction model in the form of an ensemble of weak prediction models, typically decision trees (Mayr et al., 2014). It trains many models sequentially. Each new model gradually minimizes the loss function of the whole system using gradient descent. The learning procedure consecutively fits new models to provide a more accurate estimate of the response variable. We selected decision trees with tunable hyper-parameter as base learners for our GB classifier. The GB is used to construct new base learners with maximum correlation with negative gradient of the loss function, associated with the whole ensemble.

AdaBoost (AB) is a meta-learning algorithm used to build a weak classifier iteratively on others according to the performance of the previous weak classifiers (Feng et al., 2005). The AB algorithm can be used for both classification and regression problems. The AB fits a sequence of weak learners on differently weighted training data. The process begins with prediction of the original data set and gives equal weight to each observation. If prediction is incorrect using the first learner, a higher weight is given to observation. Continuing its iterative process, the AB adds learners until a limit is reached in the number of models or accuracy. We used decision stumps (1-layer decision trees) as base learners for our AB classifier, however any machine learning algorithm can be used as base learner if it accepts weight on training data set.

Each of the dataset described in the previous section was used to train each machine learning algorithm using stratified K-Fold cross validation (CV) [see next section Cross Validation (CV)]. Grid search was conducted to determine an optimal set of hyper-parameters using the training data only. Here is the list of hyper-parameters we tuned for each of the four traditional machine learning algorithms. **SVM**: C (1e-3, 1e-2, 0.1, 1, 10), kernel (linear, rbf, poly), and gamma (1e-3, 1e-2, 0.1, 1, 10); **RF**: n_estimators (10, 20, 30, 50, 80, 100), max_depth:np.arange (1, 6, 8), min_samples_split (2, 3, 4, 5), min_samples_leaf (1, 2, 3), and max_features (0.5, log2, auto, 1.0); **GB**: n_estimators (20, 50, 80, 100), learning_rate (0.01, 0.1, 1.), max_depth:np.arange (1, 4), min_samples_split (2, 3, 4), min_samples_leaf (1, 2), and max_features (0.5, log2, auto, 1.0); **AB**: n_estimators (20, 50, 80, 100), and learning_rate (0.01, 0.1, 1.). For the definition of each parameter, the readers can refer to *Scikit-learn* (Pedregosa et al., 2011).

#### Feature Extraction and Selection

While machine learning algorithms can be developed to deal with a large number of features, such classifiers tend to have lower discriminative power and lower generalization capabilities. Both principal component analysis (PCA) (García-Gil et al., 2018) and feature selection (Li et al., 2017) were used to reduce the number of features in the dataset. In this study, we tested a few feature selection methods including the SelectKBest (SKB) module from *Scikit-learn* with *f_classif* as the metric (Pedregosa et al., 2011), Sequential Forward Selection (SFS), and Sequential Backward Selection (SBS).

#### Cross Validation (CV)

Cross validation (CV) with stratified K-Fold was used to evaluate the predictive models. For the stratified K-Fold CV, data was divided into 5 disjoint subsets with consistent ratios between patient and control in each fold. Eighty percent (80%) of the data was used in training and 20% of the data was used for testing in each fold. Note that SMOTE over-sampling, PCA, feature selection, and hyper-parameter grid search were all performed in each fold on the training data only. Leave-one-out CV with default parameters from each algorithms (without grid search) produced similar results (data not shown).

### Artificial Neural Networks (ANNs)

A perceptron is a mathematical model of a biological neuron, which is the basic computing unit for artificial neural networks (ANNs). An ANN in its simplest form has only three layers: an input layer, an output layer, and a hidden layer. Deep learning is an ANN with multiple hidden layers. A perceptron can take in a few inputs, each of which has a weight to signify how important it is, and generate an output. A deep learning system can self-teach to learn from data by filtering information through multiple hidden layers which mimicking the human brain in several ways. Deep learning networks have been developed to solve many real-world complex problems (LeCun et al., 2015).

A multilayer perceptron (MLP) is a class of feedforward ANN which often applied to supervised learning problems and uses back-propagation to adjust the weights for training. MLP is commonly used in situations where no analytical solution exists. MLP is very popular in pattern recognition systems and for interpolation and processing massive digital images. It has been used for AD detection with structural MRI images and other medical images from various types of imaging modalities (Santos et al., 2008; Joshi et al., 2010; Tufail et al., 2012).

In MLP, learning involves updating parameters including weights and biases. Training can be broken into three main steps: forward-propagation, error/loss calculation, and back-propagation. In the forward-propagation, we take an input *x* and multiply it with a weight *w* and add bias *b* as in equation (1):


(1) $$\begin{array}{r} y=\;\overset{n}{\underset{i=1}{\sum }}x_{i}w_{i}+b \end{array}$$

Where *n* denotes the input count, *x* denotes the vector of inputs, *w* denotes weights, and *b* denotes the bias. Then, the activation function is applied on the output result *y*. The target output (label) is known, and therefore can be compared against the predicted output to compute the loss. A common choice for a loss function in a binary classification task is a binary cross-entropy that can be represented as:


(2) $$\begin{array}{r} Loss=\;-y\log\left(p\right)+\left(1-y\right)\log\left(1-p\right) \end{array}$$

where *y* denotes the actual value and *p* denotes the predicted probability. Lastly, after calculating the loss, we back-propagate the loss and update the weights by using an optimizer that seeks to minimize the loss function. Weight update is performed according to the formula:


(3) $$\begin{array}{r} w\leftarrow w-\eta \frac{\partial E}{\partial w} \end{array}$$

where *w* is the weight, η is the learning rate, and $\frac{\partial \text{E}}{\partial \text{w}}$ represents the partial derivatives of the error function *E*. The partial derivative of the error function with respect to the weight can be calculated using the chain rule as follows:


(4) $$\begin{array}{r} \frac{\partial E}{\partial w}=\frac{\partial E}{\partial y_{i}}.\frac{\partial y_{i}}{\partial x_{i}}.\frac{\partial x_{i}}{\partial w} \end{array}$$

where *y*_*i*_ is the *i-th* neuron in the output layer and *x*_*i*_ is the *i-th* neuron in the input layer. Depending on the utilized loss function, the process may also involve calculating the partial derivatives at each node, adding them to the chain rule, and calculating the product of partial derivatives at each node to obtain the value of $\frac{\partial \text{E}}{\partial \text{w}}$. In implementation, a training is stopped once a convergence criteria is reached. This means a minima is reached (though this may be a local minima rather than a global minimum). A training may also be stopped after a number of epochs, or a number of passes over the training data.

In this study, we developed a fully connected MLP network for AD classification using data from neuropsychological tests and a simple 5.5-min cognitive task. We used Rectified Linear Unit (ReLU) as the activation function for the input and hidden layers. The function can be written as:


(5) $$\begin{array}{r} f\left(x\right)=max\left(0,x\right) \end{array}$$

We used the sigmoid function as the activation function for the output layer to obtain output between 0 and 1 for prediction of probabilities. The function can be written as:


(6) $$\begin{array}{r} f\left(x\right)=\;\frac{1}{1+e^{-x}} \end{array}$$

Adaptive moment estimation (Adam) (Kingma and Ba, 2015), an adaptive learning rate optimization algorithm designed specifically for training deep neural networks, was used as an optimizer. Binary cross entropy tunable with class weights was used as the loss function, in order to penalize more on the Type II errors. Due to the limited number of samples, our optimization was performed in a stochastic fashion to get the best performance: the batch size was set to 1 and the samples were shuffled before each epoch began. The maximum number of iterations was set to 250. Both the L1 and L2 regularization were added to each layer to constrain overfitting. Early stopping and learning rate shrinkage (with a minimum learning rate of 5 × 10^−4^) were performed on monitoring the validation loss function. We performed hyper-parameter tuning with different class weight ratios and probability threshold to improve the sensitivity of the proposed model. A 5-folds CV was constructed by using 20% as the testing set; the rest 80% split into the training and validation sets. The hyper-parameter tuning was performed on the training dataset using the sensitivity on the validation set as the metric. Similar results can be obtained by using the SMOTE over-sampling with more balanced class weights and probability thresholds.

### Performance Evaluation

Three performance measurements were used to evaluate the performance of each model: sensitivity, specificity, and accuracy (equations 7, 8, and 9, respectively).


(7) $$\begin{array}{r} \text{Sensitivity}=\frac{TP}{TP\;+FN} \end{array}$$

(8) $$\begin{array}{r} \text{Specificity}=\frac{TN}{TN\;+FP} \end{array}$$

(9) $$\begin{array}{r} \text{Accuracy}=\frac{TP\;+TN}{TP\;+TN\;+FP\;+FN} \end{array}$$

where: TP, True Positives; TN, True Negatives; FN, False Negatives; FP, False Positives. Based on the predicted probabilities of each subject, we also plotted the Receiver Operating Characteristic (ROC) curve and calculated the corresponding Area Under the Curve (AUC).

## Results

### Traditional Machine Learning Algorithms

Supplementary Table 1 summarizes the classification performance for four machine learning algorithms in terms of sensitivity, specificity, and accuracy using each dataset (NP, IOR_trial_, IOR_cond_, NP + IOR_trial_, and NP + IOR_cond_) and a 5-folds CV. The result from using either all features in each dataset or PCA with 90% of total variation were compared. Supplementary Table 2 is similar to Supplementary Table 1 but, in addition, applying SMOTE over-sampling technique to each dataset before training.

Supplementary Table 3 shows the best model performance for each machine learning algorithm with a feature selection technique (SKB or SFS or SBS). The numbers of features used in each algorithm were also reported which were selected based on the highest sensitivities in each model.

**Figure 2:**
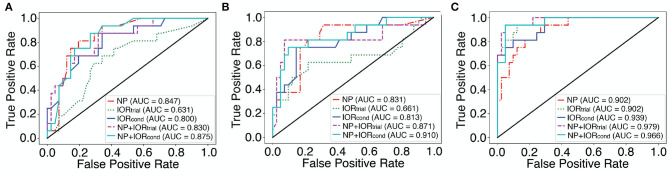
The ROC curves for the best classifiers selected by the highest sensitivity for each dataset with traditional machine learning algorithms and with MLP. See Table 2, Supplementary Tables 1-3 for the specific algorithms and parameters used for these “best” classifiers (shown in **bold** and ***italics*** font). **(A)** Traditional machine learning algorithms with all features and PCA (without and with SMOTE over-sampling). **(B)** Traditional machine learning algorithms with features selection (without and with SMOTE over-sampling). **(C)** The ROC curves for each dataset with MLP using the demographically comparable dataset. The AUC score is shown in the legend box.

Figure 2 shows the ROC curves for the best classifiers for each dataset in which the best traditional machine learning algorithm without SMOTE and with SMOTE were plotted in Figures 2A,B, respectively. Note that the best classifier selected for each dataset can be obtained from Supplementary Tables 1-3.

The classification performance is comparable between the five datasets across algorithms and techniques (Supplementary Table 4). Combining neuropsychological and cognitive data together (NP + IOR_trial_, and NP + IOR_cond_) slightly improved classification accuracy over NP only, but the difference was not significant (Supplementary Tables 1-4).

**Table 2:** Multilayer perceptron (MLP) classification performance using the demographically comparable dataset.

**Datasets**	**Probability threshold**	**Class weight**	**SEN%**	**SPE%**	**ACC ± std%**
NP	0.5	1:1	62.50	87.8	80.7 ± 7.76
	***0.4***	***1:1.17***	***75.00***	***87.8***	***84.21 ± 4.91***
IOR_trial_	0.5	1:1	75.00	95.12	89.47 ± 6.17
	***0.5***	***1:1.5***	***81.25***	***95.12***	***91.23 ± 4.28***
IOR_cond_	0.5	1:1	68.75	97.56	89.47 ± 3.12
	***0.4***	***1:1.5***	***81.25***	***90.24***	***87.72 ± 3.12***
NP + IOR_trial_	0.5	1:1	75.00	95.12	89.47 ± 7.5
	***0.5***	***1:1.1***	***87.50***	***95.12***	***92.98 ± 4.28***
NP + IOR_cond_	0.5	1:1	87.50	97.56	94.74 ± 6.75
	***0.5***	***1:1.5***	***93.75***	***92.68***	***92.98 ± 6.33***

*The sensitivity (SEN), specificity (SPE), accuracy (ACC), and standard deviation of the accuracy (std) for each dataset were calculated from 5-fold CV using the default setting for class weight (1:1) and threshold (0.5). The performance with the optimal hyper-parameter tuning for each dataset is shown in **bold** font (optimal values for class weight and threshold). IOR, inhibition of return; IOR_trial_, the reaction time and responses of each trial; IOR_cond_, the mean accuracy of all trials, mean accuracy of responded trials, and mean reaction time of each condition (Figure 1); NP, neuropsychological test scores (Table 1)*.

### Multilayer Perceptron (MLP)

The MLP classification performance with each dataset is shown in Table 2. The mean classification accuracy using the conventional choice of the class weight (1:1) and threshold (0.5) is shown, along with the model performance with an optimal setting for each dataset (shown in **bold** font). Each optimal setting was determined by tuning class weight and binarizing threshold in neural network to improve the sensitivity while maintaining accuracy. The comparative ROC curves for each dataset in Table 2 are plotted in Figure 2C.

The MLP classification performance with the 5-min cognitive task (IOR) data is noteworthy, especially with the NP + IOR_cond_ dataset (a combination of summarized scores from neuropsychological tests and the IOR task), which resulted in a high performance, suggesting that deep learning with behavioral data from certain cognitive task(s) (such as spatial IOR task tested here) may be useful to assist early AD detection/diagnosis.

The MLP classification performance using the entire study sample dataset is shown in Supplementary Table 5. A direct comparison between the best traditional machine learning and deep learning methods is concluded in Table 3 (a summary of Table 2, Supplementary Tables 1-3).

**Table 3:** A direct comparison between the best traditional machine learning and deep learning methods (a summary of Table 2, Supplementary Tables 1-3).

**Datasets**	**Method**	**SEN%**	**SPE%**	**ACC ± std%**
NP	RF	87.5	70.73	75.61 ± 5.76
	MLP	75.00	87.8	84.21 ± 4.91
IOR_trial_	AB	62.5	78.05	73.48 ± 9.75
	MLP	81.25	95.12	91.23 ± 4.28
IOR_cond_	RF	75	80.49	78.79 ± 9.25
	MLP	81.25	90.24	87.72 ± 3.12
NP + IOR_trial_	GB	81.25	92.68	89.55 ± 3.12
	MLP	87.50	95.12	92.98 ± 4.28
NP + IOR_cond_	SVM	87.5	82.93	84.09 ± 3.91
	MLP	93.75	92.68	92.98 ± 6.33

In addition, using the scores from the ADAS-Cog test – one of most commonly used neuropsychological tests in MCI/AD research and clinical trials (as a primary cognitive outcome), the specificity and sensitivity to distinguish MCI/AD patients from controls were 95.1 and 56.3% with a cut-off score of 12 (Chu et al., 2000), respectively, or 78.1 and 66.8% if a cut-off score of 10 was used (Nogueira et al., 2018).

## Discussion

Using traditional machine learning algorithms, the classification based on the 5-min cognitive task (IOR) achieved a performance comparable to the classification based on a neuropsychological test battery (which takes ~75–85 min to administer), and the classification performance was slightly improved when both sets of data (IOR and neuropsychological tests) were used. Deep learning (MLP) outperformed traditional machine learning algorithms with IOR data (either alone or together with neuropsychological data), in particularly, a high performance (~90% sensitivity and ~90% specificity) was obtained when MLP was applied to a combination of summarized scores from neuropsychological tests and the 5-min IOR task.

Using standard neuropsychological data (NP Dataset), the classification performance is comparable to other previous studies with traditional machine learning algorithms and standard neuropsychological data (Lemos et al., 2012; Williams et al., 2013; Grassi et al., 2019; Lee et al., 2019a). Similar performance was also obtained using the summarized performance data of each condition from the 5-min spatial IOR cognitive task (IOR_cond_ Dataset), suggesting that cognitive tasks such as spatial IOR might provide useful information to assist MCI/AD diagnosis. However, when the raw IOR data (i.e., response and reaction time of each individual trial) (IOR_trial_ Dataset) was used, traditional machine learning algorithms struggled with low sensitivities, suggesting that traditional machine learning algorithms might not be the best tool to extract “diagnostic” information from individual trials of a cognitive task—likely due to a high variance in responses to individual trials—and traditional machine learning algorithms might benefit greatly from “*guided*” feature reductions [i.e., by averaging responses from multiple trials of the same experimental condition (e.g., IOR_cond_ Dataset)].

Deep learning is gaining popularity in medical application, including AD. In the present study, deep learning (MLP) performed comparably to the four traditional machine learning algorithms when the standard neuropsychological data was used. Moreover, MLP with either of the two IOR datasets (IOR_trial_ or IOR_cond_) consistently outperformed traditional machine learning algorithms with either IOR dataset as well as deep learning with standard neuropsychological data, suggesting that: (i) cognitive tasks such as spatial IOR could produce rich information useful for MCI/AD diagnosis; and (ii) the rich but complex and non-linear information can be reliably extracted/captured by the deep learning algorithms (but might be difficult for traditional machine learning algorithms). It is noteworthy to point out that spatial IOR effects are robust and resistant to practice (Müller and von Mühlenen, 1996; Pratt and McAuliffe, 1999), making it an ideal tool in evaluating disease progression in longitudinal studies and effects of novel interventional treatments; and the spatial IOR task is simple and easy to understand, making it an ideal task in implement in clinical practice and research. Furthermore, combining IOR data and standard neuropsychological data further improved classification performance of MLP, with a sensitivity around 90% and a specificity above 90%, which—if independently verified in larger cohorts—has potential clinical implication.

The present study has several limitations. First, the sample size is small and there are much fewer MCI/AD patients than CN, which might contribute to the low sensitivity as well as the unsatisfying performance with traditional machine learning algorithms. To control for the imbalanced classes in deep learning analysis, we examined the performance with higher class weights for AD samples as well as lower probability thresholds that favor the MCI/AD class to improve sensitivity. The standard deviations from the 5-folds CV are low, showing a low possibility of overfitting of our artificial neural network. This is a result of using appropriate regularization and other previously described methods. Second, due to the small sample size, MCI and AD patients were combined together; this is suboptimal due to known differences between MCI and AD as well as the clinical significance of distinguishing between MCI and AD. Third, deep learning performs better with large training data sets, thus the classification performance of deep learning with the NP + IOR_cond_ (or NP + IOR_trial_) must be verified and validated by independent and larger studies. We ran an exploratory analysis using the data from the 21 excluded subjects (12 MCI/AD and 9 CN). That is, during each fold in MLP, the classifiers were also applied to the excluded subjects. There was a hit in classification performance. For instance, for NP + IOR_cond_ Dataset, the specificity remained high at 100%, but the sensitivity, however, was dropped to 66.67%. This is interesting as the excluded MCI/AD patients were either too old (>80 y.o.) or at more advanced stage of disease (i.e., failure to perform the simple IOR task due to time constraints). Indeed, a close inspection revealed that the low sensitivity was driven by four MCI/AD patients (AD, *n* = 2; MCI, *n* = 2), who were consistently misclassified as CN. All of them were older than 80 y.o. and performed high on the spatial IOR cognitive task, or the neuropsychological tests (except memory, for which three of them performed poorly), or both, suggesting that behavioral markers/features in the sixteen MCI/AD patients included in the main analysis (only three of them were older than 74 y.o.) might be different from the markers in the four relatively high performance older MCI/AD patients (Koedam et al., 2010; Ye et al., 2012). That is, visuospatial attention as measured by spatial IOR is more likely being affected in relatively young MCI/AD patients, whereas memory is more likely affected in MCI/AD patients who are at more advanced stage of age (Koedam et al., 2010; Ye et al., 2012). This is confirmed by a high classification performance when the training sample included some of the oldest MCI/AD patients (>80 y.o.) (Supplementary Table 5). In addition, we tested our methods to a large and independent dataset from ADNI (http://adni.loni.usc.edu/) and obtained encouraging classification performance using neuropsychological data (Almubark et al., 2020). However, spatial IOR data is not available in the ADNI database, thus the prediction power of double-cue spatial IOR task needs to be validated in future studies with a diverse and large sample.

In summary, most previous machine learning studies have focused on brain imaging data. With the readily availability and lower cost of behavioral data, here we investigated the classification performance of MCI/AD vs. CN using traditional machine learning algorithms and deep learning with behavioral data (from standard neuropsychological tests, a specific cognitive task, or both). Deep learning with a combination of standard neuropsychological data and cognitive task (IOR) produced a classification performance with ~90% sensitivity and ~90% specificity, which may be clinically meaningful and supports the collection of simple cognitive task(s) data in future clinical studies. However, due to the small sample size in this study, the conclusion should be taken with caution, and future studies with larger samples are needed to verify, validate, and improve upon the techniques presented in the study, i.e., to exploit public database from the large cohorts in which both standard neuropsychological tests and cognitive task(s) were administered, or to develop a mobile app to collect spatial IOR data from a large and diverse sample of participants.

## Data Availability Statement

The raw data supporting the conclusions of this article will be made available by the authors, without undue reservation.

## Ethics Statement

The studies involving human participants were reviewed and approved by Georgetown University IRB. The patients/participants provided their written informed consent to participate in this study.

## Author Contributions

IA and L-CC analyzed the data. IA, L-CC, and XJ wrote the manuscript. KS and RT revised the manuscript. RT provided clinical diagnosis of MCI and AD patients. XJ conceptualized the study, designed the behavioral experiment, and supervised the behavioral data collection. All authors reviewed the final manuscript.

## Funding

This study was funded by the Alzheimer's Drug Discovery Foundation (XJ) and the BrightFocus Foundation (XJ). The authors would also like to thank Qassim University and the Deanship of Scientific Research for their support and funding the publication.

## Conflict of Interest

The authors declare that the research was conducted in the absence of any commercial or financial relationships that could be construed as a potential conflict of interest.
