# Corrigendum: A 5-min Cognitive Task With Deep Learning Accurately Detects Early Alzheimer's Disease

**DOI:** 10.3389/fnagi.2022.879453

**Published:** 2022-03-17

**Authors:** Ibrahim Almubark, Lin-Ching Chang, Kyle F. Shattuck, Thanh Nguyen, Raymond Scott Turner, Xiong Jiang

**Affiliations:** ^1^Department of Electrical Engineering and Computer Science, Catholic University of America, Washington, DC, United States; ^2^Department of Information Technology, College of Computer, Qassim University, Buraydah, Saudi Arabia; ^3^Department of Neuroscience, Georgetown University Medical Center, Washington, DC, United States; ^4^Department of Neurology, Georgetown University Medical Center, Washington, DC, United States

**Keywords:** Alzheimer's disease, machine learning, artificial neural networks, inhibition of return, neuropsychological test

In the original article, we neglected to include the funders “Qassim University and the Deanship of Scientific Research to Ibrahim Almubark.” The Funding Statement has been updated to include “The authors would also like to thank Qassim University and the Deanship of Scientific Research for their support and funding the publication.”

In the published article, there was an error regarding the affiliations for “Ibrahim Almubark.” As well as having affiliation 1, they should also have affiliation 2.

The authors apologize for these errors and state that they do not change the scientific conclusions of the article in any way. The original article has been updated.

## Publisher's Note

All claims expressed in this article are solely those of the authors and do not necessarily represent those of their affiliated organizations, or those of the publisher, the editors and the reviewers. Any product that may be evaluated in this article, or claim that may be made by its manufacturer, is not guaranteed or endorsed by the publisher.

