# Supplementary material for: A 5-min Cognitive Task With Deep Learning Accurately Detects Early Alzheimer's Disease
Source: Front Aging Neurosci. 2020 Dec 3;12:603179. doi: 10.3389/fnagi.2020.603179 (PMC7744695; doi:10.3389/fnagi.2020.603179)
Supplement: Supplementary file 1 [file Table_1.pdf]

A five-minute cognitive task with deep learning accurately detects early Alzheimer's disease

SUPPLEMENTARY MATERIAL

Ibrahim Almubark[a], Lin-Ching Chang[a], Kyle F. Shattuck[b], Thanh Nguyen[a], Raymond Scott Turner[c], and Xiong Jiang[b*]

[a]*Department of Electrical Engineering and Computer Science, Catholic University of America, Washington, DC, USA;* [b]*Department of Neuroscience &* [c]*Department of Neurology, Georgetown University Medical Center, Washington, DC, USA*

Table S1. Classification performance (sensitivity (SEN), specificity (SPE), accuracy (ACC), and standard deviation of the accuracy (std)) with the four traditional machine learning algorithms with all features and features extraction with PCA. IOR, inhibition of return; IOR$_{trial}$, the reaction time and responses of each trial; IOR$_{cond}$, the mean accuracy of all trials, mean accuracy of responded trials, and mean reaction time of each condition (Fig. 1); NP, neuropsychological test scores (Table 1).

Datasets	Algorithms	All Features			PCA		
		SEN%	SPE%	ACC ± std%	SEN%	SPE%	ACC ± std%
NP	SVM	56.25	90.24	80.76 ± 2.94	50	90.24	79.09 ± 3.34
	RF	56.25	90.24	80.91 ± 7.88	37.5	85.37	71.97 ± 6.39
	GB	68.75	85.37	81.06 ± 11.97	43.75	87.8	75.45 ± 6.65
	AB	62.5	80.49	75.61 ± 11.03	50	87.8	77.12 ± 14.8
IOR$_{trial}$	SVM	50	90.24	78.79 ± 11.66	25	90.24	72.12 ± 7.66
	RF	43.75	92.68	78.79 ± 7.25	18.75	92.68	71.97 ± 2.79
	GB	31.25	80.49	66.67 ± 10.24	18.75	92.68	72.12 ± 7.66
	AB	***56.25***	***73.17***	***68.33 ± 10.52***	31.25	80.49	66.52 ± 10.39
IOR$_{cond}$	SVM	43.75	92.68	78.79 ± 7.25	43.75	85.37	73.64 ± 5.86
	RF	50	95.12	81.97 ± 12.97	50	85.37	75.45 ± 8.48
	GB	43.75	95.12	80.3 ± 12.12	56.25	92.68	82.27 ± 6.01
	AB	43.75	95.12	80.3 ± 10.67	50	82.93	73.64 ± 7.88
NP + IOR$_{trial}$	SVM	68.75	82.93	79.09 ± 12.46	25	92.68	73.94 ± 8.91
	RF	25	92.68	74.09 ± 9.82	31.25	92.68	75.61 ± 5.76
	GB	25	87.8	70.3 ± 6.47	43.75	87.8	75.15 ± 9.13
	AB	43.75	92.68	78.94 ± 4.24	31.25	85.37	69.85 ± 9.89
NP + IOR$_{cond}$	SVM	50	90.24	78.94 ± 4.24	50	87.8	77.27 ± 3.8
	RF	37.5	92.68	77.12 ± 4.56	68.75	85.37	80.76 ± 6.46
	GB	56.25	87.8	78.94 ± 11.82	43.75	90.24	77.12 ± 4.56
	AB	56.25	87.8	79.09 ±12.46	68.75	82.93	78.79 ± 4.98

Table S2. Classification performance (sensitivity (SEN), specificity (SPE), accuracy (ACC), and standard deviation of the accuracy (std)) with the four traditional machine learning algorithms with all features and features extraction with PCA using SMOTE over-sampling. IOR, inhibition of return; IOR_{trial}, the reaction time and responses of each trial; IOR_{cond}, the mean accuracy of all trials, mean accuracy of responded trials, and mean reaction time of each condition (Fig. 1); NP, neuropsychological test scores (Table 1).

Datasets	Algorithms	All Features - SMOTE			PCA - SMOTE		
		SEN%	SPE%	ACC ± std%	SEN%	SPE%	ACC ± std%
NP	SVM	37.5	75.61	65 ± 8.81	62.5	58.54	59.39 ± 9.8
	RF	***75***	***82.93***	***80.61 ± 7***	62.5	70.73	68.64 ± 10.98
	GB	68.75	82.93	78.94 ± 7.15	68.75	80.49	77.12 ± 9.32
	AB	62.5	73.17	70.45 ± 7.76	62.5	73.17	70.3 ± 8.35
IOR_{trial}	SVM	37.5	87.8	73.64 ± 5.39	25	92.68	73.64 ± 7.88
	RF	37.5	82.93	70 ± 9.69	43.75	80.49	70.15 ± 14.72
	GB	37.5	87.8	73.48 ± 8.52	18.75	75.61	59.7 ± 18.52
	AB	18.75	85.37	66.52 ± 10.89	25	65.85	54.55 ± 17.95
IOR_{cond}	SVM	43.75	73.17	64.55 ± 10.81	75	65.85	68.18 ± 12.57
	RF	62.5	82.93	76.97 ± 13.08	68.75	68.29	68.03 ± 10.03
	GB	56.25	82.93	75.15 ± 11.03	50	73.17	67.12 ± 12.76
	AB	62.5	75.61	71.67 ± 11.03	***75***	***65.85***	***68.33 ± 8.81***
NP + IOR_{trial}	SVM	37.5	90.24	75.3 ± 7.07	37.5	85.37	72.12 ± 9.58
	RF	***68.75***	***87.8***	***82.58 ± 7.67***	50	80.49	71.82 ± 10.57
	GB	43.75	80.49	69.85 ± 12.81	37.5	73.17	63.18 ± 10.32
	AB	50	87.8	76.82 ± 12.65	31.25	85.37	70.15 ± 11.83
NP + IOR_{cond}	SVM	***68.75***	***90.24***	***83.94 ± 8.87***	62.5	80.49	75.61 ± 5.76
	RF	56.25	90.24	80.61 ± 4	50	80.49	72.27 ± 9.04
	GB	62.5	90.24	82.42 ± 5.32	56.25	82.93	75.45 ± 11.06
	AB	62.5	85.37	78.94 ± 8.58	68.75	80.49	77.27 ± 6.5

Table S3. Best combination of feature selection techniques and the number of features selected for each algorithm and dataset with the highest sensitivity without and with SMOTE over-sampling and with the four traditional machine learning algorithms. The classification performance (sensitivity (SEN), specificity (SPE), accuracy (ACC), and standard deviation of the accuracy (std)) is also tabulated. IOR, inhibition of return; IOR_{trial}, the reaction time and responses of each trial; IOR_{cond}, the mean accuracy of all trials, mean accuracy of responded trials, and mean reaction time of each condition (Fig. 1); NP, neuropsychological test scores (Table 1).

Datasets	Algorithms	Without SMOTE					With SMOTE				
		Method	Features	SEN %	SPE %	ACC ± std %	Method	Features	SEN %	SPE %	ACC ± std%
NP	SVM	SKB	8	62.5	90.24	82.42 ± 5.32	SFS	2	81.25	82.93	82.73 ± 7.13
	RF	SKB	4	68.75	90.24	84.39 ± 8.32	***SBS***	***2***	***87.5***	***70.73***	***75.61 ± 5.76***
	GB	SKB	8	62.5	85.37	79.09 ± 8.48	SFS	4	81.25	78.05	79.09 ± 8.48
	AB	SFS	2	68.75	85.37	80.45 ± 7.15	SFS	2	81.25	70.73	73.64 ± 5.39
IOR_{trial}	SVM	SKB	90	50	95.12	82.27 ± 9.58	SBS	90	56.25	70.73	66.67 ± 13.2
	RF	SBS	90	37.5	92.68	77.12 ± 7.34	SKB	70	56.25	75.61	69.7 ± 11.62
	GB	SBS	10	50	82.93	73.48 ± 10.28	SFS	50	56.25	70.73	66.67 ± 6.23
	AB	SBS	10	56.25	80.49	73.18 ± 19.37	***SFS***	***30***	***62.5***	***78.05***	***73.48 ± 9.75***
IOR_{cond}	SVM	SKB	6	56.25	95.12	84.39 ± 6.01	SFS	12	68.75	73.17	71.67 ± 12
	RF	SFS	14	56.25	92.68	82.12 ± 8.47	***SBS***	***14***	***75***	***80.49***	***78.79 ± 9.25***
	GB	SBS	12	62.5	90.24	82.27 ± 6.01	SBS	4	75	68.29	70.45 ± 10.7
	AB	SBS	8	56.25	92.68	82.12 ± 9.98	SBS	16	68.75	85.37	80.45 ± 7.15
NP + IOR_{trial}	SVM	SKB	50	56.25	95.12	84.24 ± 6.27	SKB	70	68.75	85.37	80.45 ± 9.17
	RF	SBS	90	50	92.68	80.76 ± 6.46	SBS	10	75	82.93	80.76 ± 6.46
	GB	SFS	70	56.25	80.49	73.64 ± 5.86	***SFS***	***90***	***81.25***	***92.68***	***89.55 ± 3.12***
	AB	SKB	70	62.5	82.93	76.97 ± 11.23	SFS	90	75	75.61	75.15 ± 10.2
NP + IOR_{cond}	***SVM***	SBS	14	68.75	85.37	80.61 ± 4	***SFS***	***12***	***87.5***	***82.93***	***84.09 ± 6.91***
	RF	SKB	12	56.25	92.68	82.27 ± 10.11	SFS	16	75	90.24	85.76 ± 9.17
	GB	SKB	22	75	87.8	84.39 ± 11.17	SKB	24	75	85.37	82.58 ± 9.01
	AB	SBS	12	62.5	97.56	87.42 ± 7.35	SKB	16	75	87.8	84.24 ± 6.27

Table S4. Comparisons of classification accuracy between different datasets: NP vs. IOR_{trial}, NP vs. IOR_{cond}, NP vs. NP + IOR_{trial}, and NP vs. NP + IOR_{cond}. One-way ANOVA test was used, and *p*-values are presented below without correction for multiple comparisons. IOR, inhibition of return; IOR_{trial}, the reaction time and responses of each trial; IOR_{cond}, the mean accuracy of all trials, mean accuracy of responded trials, and mean reaction time of each condition (Fig. 1); NP, neuropsychological test scores (Table 1).

Technique	NP vs. IOR_{trial}	NP vs. IOR_{cond}	NP vs. NP + IOR_{trial}	NP vs. NP + IOR_{cond}
All Features	79.59 vs. 73.15	79.59 vs. 80.34	79.59 vs. 75.61	79.59 vs. 78.52
p-values	0.12	0.63	0.16	0.48
All Features - SMOTE	73.75 vs. 70.91	73.75 vs. 72.09	73.75 vs. 76.14	73.75 vs. 81.48
p-values	0.51	0.73	0.62	0.09
PCA	75.91 vs. 70.68	75.91 vs. 76.25	75.91 vs. 73.64	75.91 vs. 78.49
p-values	0.04	0.90	0.30	0.19
PCA - SMOTE	68.86 vs. 64.51	68.86 vs. 67.92	68.86 vs. 69.32	68.86 vs. 75.15
p-values	0.48	0.80	0.92	0.15
Feature Selection	81.59 vs. 76.51	81.59 vs. 82.73	81.59 vs. 78.90	81.59 vs. 83.67
p-values	0.08	0.41	0.34	0.31
Feature Selection - SMOTE	77.77 vs. 69.13	77.77 vs. 75.34	77.77 vs. 81.48	77.77 vs. 84.17
p-values	0.02	0.48	0.34	0.02

Table S5. Multilayer perceptron (MLP) classification performance using the entire study sample dataset. The sensitivity (SEN), specificity (SPE), accuracy (ACC), and standard deviation of the accuracy (std) for each dataset were calculated from 5-fold CV using the default setting for class weight (1:1) and threshold (0.5). The performance with the optimal hyper-parameter tuning for each dataset is shown in **bold** font (optimal values for class weight and threshold). IOR, inhibition of return; IOR_{trial}, the reaction time and responses of each trial; IOR_{cond}, the mean accuracy of all trials, mean accuracy of responded trials, and mean reaction time of each condition (Fig. 1); NP, neuropsychological test scores (Table 1).

Datasets	Probability Threshold	Class Weight	SEN%	SPE%	ACC ± std%
NP	0.5	1:1	75.00	96.00	88.46 ± 7.32
	**0.4**	**1:1.17**	**85.71**	**90.00**	**88.46 ± 4.78**
IOR_{trial}	0.5	1:1	71.43	98.00	88.46 ± 8.24
	**0.3**	**1:1.1**	**92.86**	**90.00**	**91.03 ± 4.92**
IOR_{cond}	0.5	1:1	71.43	94.00	85.90 ± 2.36
	**0.4**	**1:1.5**	**85.71**	**86.00**	**85.90 ± 3.97**
NP + IOR_{trial}	0.5	1:1	82.14	98.00	92.13 ± 7.28
	**0.3**	**1:1**	**92.86**	**94.00**	**93.59 ± 3.96**
NP + IOR_{cond}	0.5	1:1	85.71	98.00	93.59 ± 6.85
	**0.4**	**1:3**	**92.86**	**96.00**	**94.87 ± 4.73**
